# Temporal dynamics of mirror-symmetry perception

**DOI:** 10.1167/18.5.10

**Published:** 2018-05-15

**Authors:** Rebecca J. Sharman, Sebastian Gregersen, Elena Gheorghiu

**Affiliations:** rebecca.sharman@stir.ac.uk; s.o.gregersen@stir.ac.uk; elena.gheorghiu@stir.ac.uk https://www.stir.ac.uk/people/19056; University of Stirling, Department of Psychology, Stirling, Scotland, UK; University of Stirling, Department of Psychology, Stirling, Scotland, UK; University of Stirling, Department of Psychology, Stirling, Scotland, UK

**Keywords:** symmetry, temporal delay, temporal integration, psychophysics, perceptual organization

## Abstract

Recent studies have suggested that temporal dynamics rather than symmetrical motion-direction contribute to mirror-symmetry perception. Here we investigate temporal aspects of symmetry perception and implicitly, its temporal flexibility and limitations, by examining how symmetrical pattern elements are combined over time. Stimuli were dynamic dot-patterns consisting of either an on-going alternation of two images (sustained stimulus presentation) or just two images each presented once (transient stimulus presentation) containing different amounts of symmetry about the vertical axis. We varied the duration of the two images under five temporal-arrangement conditions: (a) *whole patterns* in which a symmetric pattern alternated with a noise pattern; (b) *delayed halve*s—the halves of the symmetric and noise patterns were presented with temporal delay; (c) *matched-pairs—t*wo alternating images each containing equal amounts of symmetrical matched-pairs; (d) *delayed matched-pair*s—the same as arrangement (c), but with matched-pairs presented with delay; and (e) *stati*c—both images presented simultaneously as one. We found increased sensitivity in sustained compared to transient stimulus presentations and with synchronous compared to delayed matched-pairs stimuli. For the delayed conditions, sensitivity decreased gradually with longer image durations (>60 ms), prominently for the transient stimulus presentations. We conclude that spatial correlations across-the-symmetry-midline can be integrated over time (∼120 ms), and symmetry mechanisms can tolerate temporal delays between symmetric dot-pairs of up to ∼60 ms.

## Introduction

Mirror symmetry (henceforth “symmetry”) is a ubiquitous visual feature in natural images that occurs when one half of an image reflects the other about an axis. Symmetry is a salient visual feature found in both natural and man-made objects, to which the human visual system is highly sensitive. Psychophysical, computational, and brain imaging (fMRI) studies have shown that symmetry plays an important role in figure-ground segregation (Driver, Baylis, & Rafal, 1992; Machilsen, Pauwels, & Wagemans, 2009; Makin, Rampone, Wright, Martinovic, & Bertamini, 2014; Metzger, 2009), object recognition (Pashler, 1990; Vetter & Poggio, 1994; Vetter, Poggio, & Bulthoff, 1994), amodal completion (Saiki, 2000; van Lier, Vanderhelm, & Leeuwenberg, 1995), and visual search (Wolfe & Friedmanhill, 1992) and, involves an extensive network of extrastriate visual areas such as V3a, V4, V7, and LOC (Sasaki, Vanduffel, Knutsen, Tyler, & Tootell, 2005; Tyler et al., 2005). Although several recent studies have examined the contribution of simple visual attributes, such as luminance-polarity and color (Gheorghiu, Kingdom, Remkes, Li, & Rainville, 2016; Morales & Pashler, 1999; Wu & Chen, 2014), stereoscopic depth (Ishiguchi & Yakushijin, 1999; Treder & van der Helm, 2007), and motion direction (Sharman & Gheorghiu, 2017) to symmetry perception, little or nothing is known about the temporal dynamics of symmetry perception. While recent studies have suggested that temporal dynamics contribute to symmetry perception (Sharman & Gheorghiu, 2017), none have considered temporal aspects of symmetry perception in *dynamic* stimuli. In this communication, we investigate sustained and transient properties of symmetry perception about vertical axis by examining how symmetrical pattern-elements are combined over time, and whether symmetry mechanisms can tolerate temporal delays between matched elements. By studying both sustained and transient aspects of symmetry perception, one can gain insight into the temporal characteristics of the mechanisms underlying symmetry perception and, implicitly, about their temporal flexibility and limitations.

Psychophysical and neurophysiological studies indicate that temporal information plays a critical role in many visual processes such as stereoscopic depth (Gheorghiu & Erkelens, 2005a; Gheorghiu & Erkelens, 2005b; Hess, Mansouri, Thompson, & Gheorghiu, 2009), form (Eriksen & Collins, 1967; Niimi, Watanabe, & Yokosawa, 2008; Sharman & Gheorghiu, 2017), and motion (Burr, 1981; Burr & Santoro, 2001) perception. Specifically, it appears that two temporal factors are of major importance for visual perception, namely *duration* and *synchronization* (i.e., simultaneity of presentation) between corresponding or matched stimulus parts or elements. With regard to duration, many visual features, which require integration across space, can be perceived with only very short stimulus durations. For example, stereopsis (i.e., disparity-defined depth) can be perceived in random-dot stereograms with very brief presentation durations between 1 ms (Uttal, Davis, & Welke, 1994) and 60 ms (Gheorghiu & Erkelens, 2005a; Gheorghiu & Erkelens, 2005b; Uttal, Fitzgerald, & Eskin, 1975). Similarly, the perception of motion streaks requires stimulus durations of at least 77 ms (Alais, Apthorp, Karmann, & Cass, 2011). As for symmetry, this can be reliably detected at the fixation point in static stimuli presented for as short as 30 to 50 ms (Julesz, 1971; Tyler, Hardage, & Miller, 1995) although most studies of symmetry perception use stimulus durations of about 400–500 ms (Gheorghiu et al., 2016; Sharman & Gheorghiu, 2017; Wu & Chen, 2014; Wu & Chen, 2017). Using symmetric textures, Cohen and Zaidi (2013) found *temporal* thresholds for identifying the *orientation* of symmetry axis that range between 28 to 568 ms. Thus, observers can perceive symmetry even though these stimulus durations do not allow for sequential examination of individual symmetric pairs (Niimi et al., 2008; Tyler et al., 1995; Wagemans, 1995). Furthermore, Treder and van der Helm (2007) examined the interaction between symmetry detection and stereoscopic depth mechanisms by using static stimuli in which symmetrical matched-pairs were distributed either on the same or different depth planes and presented for various durations between 200 ms and 1 s. These authors reported that the efficient detection of symmetry in stereoscopic vision depends on structural correspondences within depth planes and requires longer stimulus durations, whereas symmetry for short presentation durations (200 ms) relies on monocular mechanisms.

Other studies, however, used unlimited stimulus presentations and measured reaction times for detecting symmetry in nonisoluminant patterns made of two and four colors (Morales & Pashler, 1999). Morales and Pashler (1999) found longer and less accurate responses to the four-color (2 s) than two-color (1.2 s) patterns, thus arguing that symmetry in multicolor patterns could only be detected by switching attention from one color to the next and assessing individually the symmetry for each color. In sum, the use of either briefly presented static stimuli or an unlimited stimulus presentation time allowing sequential examination of symmetrical pairs does not reflect the time period over which the visual system integrates symmetrical pairs, i.e., computes spatial correlations across the symmetry-midline over time. Instead these durations might reflect the minimum time needed to detect a perceptual change in the stimulus. Recent studies have suggested that symmetry is subject to a cumulative temporal process, where weak symmetry signals are combined over time to form a relatively stronger response (Niimi et al., 2008; Sharman & Gheorghiu, 2017). There are, however, no studies that have *directly* examined how symmetry mechanisms integrate matched-pairs across the symmetry axis over time in dynamic stimuli.

It has been suggested that when studying temporal properties, it is important to distinguish between *transient* (i.e., brief stimulus exposures in which each image is only presented once) and *sustained* (i.e., longer stimulus durations in which the images are continuously alternated) *stimulus presentations* as these two forms of presentation may be mediated by distinct underlying mechanisms (Edwards, Pope, & Schor, 1999; Gheorghiu & Erkelens, 2004; Pope, Edwards, & Schor, 1999; Schor, Edwards, & Pope, 1998). Evidence for separate sustained and transient mechanisms comes from stereo-vision domain where it has been suggested that spatially complex stimuli (e.g., dot patterns) can only be processed by the sustained system (Pope et al., 1999). For clarity, the terms *transient* and *sustained* can refer to the type of stimulus presentation, to the underlying mechanism, or to the percept. Hence, in this study we will examine symmetry perception in response to both sustained (i.e., prolonged) and transient stimulus presentations. A sustained stimulus presentation allows the visual system to integrate weak symmetry signals over time, within a specific time window, whereas a transient presentation allows for decay in the strength of the symmetry signals over time. This predicts increased sensitivity to symmetry for sustained compared to transient stimulus presentations, and for higher than lower alternation frequencies.

By studying sustained and temporal properties of symmetry perception in dynamic stimuli, one can gain insight into how symmetry mechanisms integrate matched-pairs across the symmetry axis and *across time*. Thus, one important temporal factor that can influence how a stimulus is perceived is the synchronization or simultaneity of presentation of spatially correlated or matched stimulus elements. For example, it is known that synchronization of the left and right eyes' images plays an important role in disparity-defined depth perception (Gheorghiu & Erkelens, 2005b). However, disparity-defined depth can also be perceived when one retinal image is somewhat delayed relative to the other, a phenomenon referred to as *tolerance for inter-ocular delays*. Psychophysical studies have found that the stereoscopic system can tolerate a time difference between binocularly correlated images of up to 50 ms (Gheorghiu & Erkelens, 2005b; Julesz & White, 1969; Ross & Hogben, 1974). As for symmetry perception, which requires computation of spatially matched-elements across the symmetry axis, little is known about whether symmetry mechanisms can tolerate delays between the matched pairs. Only one study by Hogben, Julesz, and Ross (1976) examined the effect of temporal delays between briefly presented matched-elements on *orientation discrimination* of the symmetry axis and reported that symmetry perception ceased with delays of ∼50–90 ms. Thus, it remains to be established how temporal delays between matched-elements are affected by the sustained and transient stimulus presentation and by changes in the amount of symmetry (i.e., strength of symmetry signals) within the temporal integration window. To test for this effect, we will use stimuli in which symmetric pairs are presented either simultaneously or with a variable time delay between spatially-matched elements. We predict that in conditions where the symmetric pairs are presented with delay, there will be a temporal limit beyond which the symmetric elements cannot be spatially correlated. Thus, by varying the temporal delay between spatially matched elements and the amount of image symmetry over time, we will examine temporal integration mechanisms for symmetry processing and their flexibility and limitations (e.g., tolerance for temporal delays between spatially-matched elements).

Several categories of computational models have been developed for detecting and localizing symmetry in an image by using either pixel-by-pixel correlations between the symmetric halves (Barlow & Reeves, 1979; Gurnsey, Herbert, & Kenemy, 1998; Pintsov, 1989), complex grouping rules based on higher-order structural correlations from which symmetry is subsequently extracted (Labonte, Shapira, Cohen, & Faubert, 1995; Pashler, 1990; Wagemans, Vangool, Swinnen, & Vanhorebeek, 1993), or early spatial mechanisms such as oriented filters to compute mirror-symmetry (i.e., symmetrical dot-pairs are detected directly by the outputs of oriented receptive fields, RFs, of various sizes; Cohen & Zaidi, 2013; Dakin & Watt, 1994; Rainville & Kingdom, 2002). However, none of these models have incorporated temporal aspects, although it is well established that RFs of cortical neurons are spatiotemporally oriented, i.e., tilt along an oblique axis in the space-time domain making them space-time inseparable; for a review see Orban (1991). Although the existing models and algorithms demonstrate that symmetry is a global image property requiring not just first-order oriented filters, but additional subsequent processing (e.g., spatial correlation of symmetrical pairs across the axis of symmetry), it remains to be determined what consequences time (i.e., duration and synchronization of matched-pairs) has on these models and on the perception of symmetry in dynamic stimuli.

**Figure 1:**
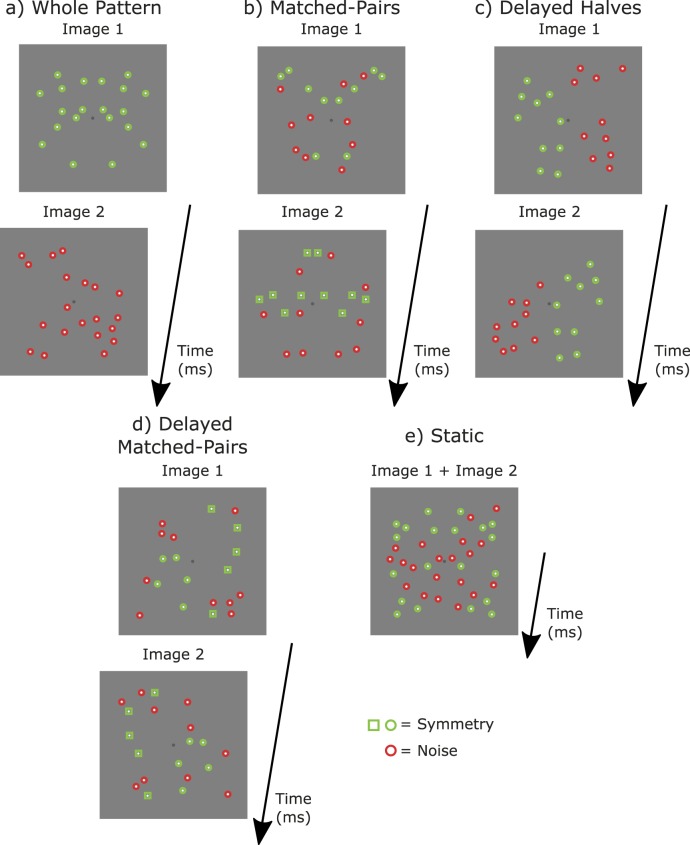
Example stimuli. Symmetrical (signal) dots are outlined in green, with matched pairs having the same shaped outline (i.e., circle or square). Random (noise) dots are outlined in red. Red and green outlines are for illustrative purposes and not present in the actual stimuli. There were five temporal-arrangement conditions: (a) Whole patterns, in which a symmetrical pattern (Image 1) is alternated with a noise pattern (Image 2). (b) Matched-pairs, in which half of the total number of symmetrical dots is presented in each image or interval (see green circles and squares). (c) Delayed halves, in which left and right halves of the symmetrical and noise patterns were presented with temporal delay, i.e., half of the symmetrical pattern is presented in each image or interval. (d) Delayed matched-pairs, which is the delayed version of (c), i.e., the matched elements are presented in different intervals as shown by the green squares and circles. Note that in both delayed conditions (c) and (d), there are no symmetrical matched-pairs in either time interval. (e) Static, in which the symmetrical and noise elements in the two images were presented simultaneously as one single static pattern which was the time average of Image 1 and Image 2.

In this study, we examine temporal properties of symmetry perception in response to *sustained and transient stimulus presentations* by using dynamic stimuli consisting of an on-going alternation of two images (i.e., sustained stimulus presentation) or only two images (i.e., transient stimulus presentation) containing varying amounts of symmetry about the vertical axis. To investigate how spatial correlations between elements across the symmetry axis is computed and integrated over time, we use patterns in which the symmetrical elements are presented either simultaneously or with temporal delay. For the simultaneous (or synchronous) presentation, stimuli consisted of two alternating patterns: a symmetrical pattern and a noise pattern (i.e., whole patterns condition; see Figure 1a and Supplementary Movie S1 for the dynamic version of the stimuli) or two patterns, each containing an equal number of symmetrical pairs (i.e., matched-pairs condition; see Figure 1b and Supplementary Movie S2). To determine the extent to which symmetry mechanisms tolerate delays, we used the same conditions as above, but with stimulus halves and matched-pairs presented with delay i.e., delayed halves (see Figure 1c and Supplementary Movie S3) and delayed matched-pairs (see Figure 1d and Supplementary Movie S4) conditions, respectively. In addition, we compare symmetry perception in dynamic stimuli with that obtained using static patterns resulting from temporal averaging of the two alternating images (Figure 1e). For all conditions, we varied the amount of symmetry and the temporal alternation rate of the two images in order to systematically examine how the perception of symmetry changes with temporal frequency. We then compare the threshold and the slope of the psychometric function for the simultaneous and delayed conditions, and for both sustained and transient stimulus presentation conditions. If symmetry is perceived in any of the delayed conditions, then this will indicate the degree to which symmetry mechanisms can tolerate temporal delays between matched pairs. Altogether, these findings will provide an in-depth characterization of the temporal aspects of symmetry mechanisms in dynamic stimuli and, implicitly, their limitations.

## Methods

### Participants

Five observers participated in the sustained presentation experiment and four observers in the transient presentation experiment. All participants had normal or corrected-to-normal vision. Observers gave their informed consent prior to participating in the study and were treated in accordance with the Helsinki Declaration (Version 6). All procedures were approved by the University of Stirling, Psychology Ethics Committee.

### Stimuli: Generation and display

Stimuli were presented on a gamma-corrected 20-in. ViewSonic Professional Series PF817 cathode ray tube (CRT) monitor (ViewSonic, Brea, CA) with spatial resolution of 1,024 × 768 and refresh rate of 85 Hz. A ViSaGe MKII stimulus generator (Cambridge Research Systems, Cambridge, UK) in Bits# mode was used to control contrast. All stimuli were presented in the center of the monitor on a midgray background with mean luminance of 47.2 cd/m^2^. Viewing distance was 52 cm. All stimuli were generated and all data were collected using PsychoPy (Peirce, 2007).

Stimuli were presented in a square window 9.034° in width and were comprised of 20 circular white dots (100% contrast) of 0.169° diameter. The symmetrical dots were positioned randomly on the left side of the stimulus area and then mirrored about the vertical axis onto the right side. Noise dots were positioned randomly such that equal numbers appeared in each stimulus half. All dots were positioned a minimum of 0.767° apart. This resulted in a stimulus dot density of 0.7 dots/deg^2^.

Stimuli were dynamic dot patterns consisting of the on-going alternation of two images containing different amounts of symmetry (i.e., sustained stimulus presentation) or two images each presented once (i.e., transient stimulus presentation). There were five temporal-arrangement conditions: (a) *whole patterns* consisting of a symmetrical pattern alternated with a noise pattern (Figure 1a); (b) *delayed halves* in which the left and right halves of the symmetrical and noise patterns were presented with temporal delay (Figure 1c); (c) *matched-pairs* consisting of two alternating symmetrical patterns each containing equal amounts of symmetrical matched-pairs (Figure 1b). Note, this does not mean that half of the dots in each image are symmetrical, but instead that half of the total number of symmetrical dots in the stimulus are in each image. For example, if the stimulus contains 16 symmetrical dots (i.e., eight pairs), then eight symmetrical dots (i.e., four pairs) would be shown in each image; (d) *delayed matched-pairs*, which is the same as arrangement (c), but with the matched-pairs presented with temporal delay (Figure 1d), and (e) *static,* in which the symmetrical and noise elements in the two images were presented simultaneously as one static pattern, which was the temporal average of the two images (Figure 1e). Note that in both delayed conditions (Figure 1c and d) there are no matched-pairs in either time interval.

For each sustained condition, the two alternating images were presented for equal amounts of time. For clarity, the term *image duration* refers to the amount of time each component image of the dynamic stimulus is shown, whereas the term *total stimulus duration* refers to the total amount of time the dynamic stimulus (i.e., the on-going alternating images) is presented on the screen. In the sustained condition, the total stimulus duration was always the same 2.35 s, while we varied the image duration of the two alternating images between 23.5 ms and 293.1 ms in six steps: 23.5, 47.1, 58.8, 117.7, 235.3, and 294.1 ms. These image durations correspond to the following temporal frequencies: 42.5, 21.3, 17, 8.5, 4.3, and 3.4 Hz, respectively, and were selected to ensure that, in the sustained stimulus presentation condition, they allow both alternating images to be presented an even number of times within the total stimulus presentation duration of 2.35 s.

In the transient presentation experiment, the individual image durations were the same as those used in the sustained presentation experiment, but each of the two images were shown only once (i.e., for one full cycle), and as a result, the total stimulus duration (i.e., image 1 and image 2 or the full cycle length) varied with the image duration. For this experiment, we varied the presentation order of the two images: Image 1 followed by Image 2 (i.e., order 1) and Image 2 followed by Image 1 (i.e., order 2).

### Procedure

A single interval forced-choice procedure was employed for both sustained and transient experiments. For the sustained presentation experiment, on each trial, the stimulus consisted of the ongoing alternation of two images corresponding to one of the five temporal-arrangement conditions (see Figure 1) and was presented for 2.353 s. In the transient presentation experiment, each image was only shown once in one of the two possible presentation orders (i.e., order 1 or order 2). The participants' task was to indicate, by a key press, whether the entire stimulus, as a whole, was symmetric or not (i.e., yes/no task). This was particularly important for conditions with longer presentation times when the two alternating images were perceived as flickering. In order to ensure that participants understood the task, they were allowed as many practice trials as necessary.

The amount of symmetry was varied in accordance with the method of constant stimuli. For each temporal-arrangement condition and each image duration (23.5, 47.1, 58.8, 117.7, 235.3, and 294.1 ms), we varied the percentage of symmetric dots in the stimulus between 0% (noise) and 100% (fully symmetric) in steps of 5% (i.e., two dots) and measured the percentage of trials in which participants perceived each stimulus as being symmetrical (i.e., percentage perceived symmetric). In each run, corresponding to each image duration, all possible levels of symmetry were presented ten times each, in random order. Each participant collected a minimum of five runs for each image duration condition (550 trials) resulting in 3,300 trials (6 image durations × 550 trials) for each temporal-arrangement condition. Given the five temporal-arrangement conditions, this resulted in 16,500 trials per participant, for the sustained presentation experiment. For the transient presentation experiment, a similar number of trials were obtained for each presentation order condition.

Since the task required participants to judge whether a stimulus is symmetrical or not by comparing it to an internal criterion/reference, there might be some effect of participant bias. Therefore, in order to decouple sensitivity to symmetry from bias for each participant and each stimulus symmetry condition, we calculated *d*′ (“*d*-prime”) values using the function PAL_SDT_1AFC_PHFtoDP from the Palamedes toolbox (http://www.palamedestoolbox.org) described in Kingdom and Prins (2016) and Prins and Kingdom (2009). This function converts proportion hits and proportion false alarm rates into *d*′ values for a one-alternative, forced-choice task.

A logistic function was fit to the percentage perceived symmetric data as a function of the percentage of symmetry signal in the stimuli, for each image duration, and each temporal-arrangement condition, in order to estimate the number of symmetric dots (or signal) required for the observer to perceive the dynamic pattern as symmetrical in 50% of the trials. For some conditions, specifically for the delayed halves and delayed matched-pairs conditions with longer image durations, participants were not able to perceive symmetry, irrespective of the number of symmetrical dot-pairs present in the stimuli. Therefore, for these conditions the logistic functions were very shallow, and it was not possible to calculate thresholds. For this reason, the slope of the logistic function (the beta *β* coefficient) was calculated as a measure relating symmetry sensitivity and symmetry signal strength: The shallower the slope (i.e., the smaller the beta coefficient), the less the participant could differentiate between the different stimulus symmetry levels.

## Results

All data and analyses are available online at http://hdl.handle.net/11667/95.

### Sustained stimulus presentation experiment

**Figure 2:**
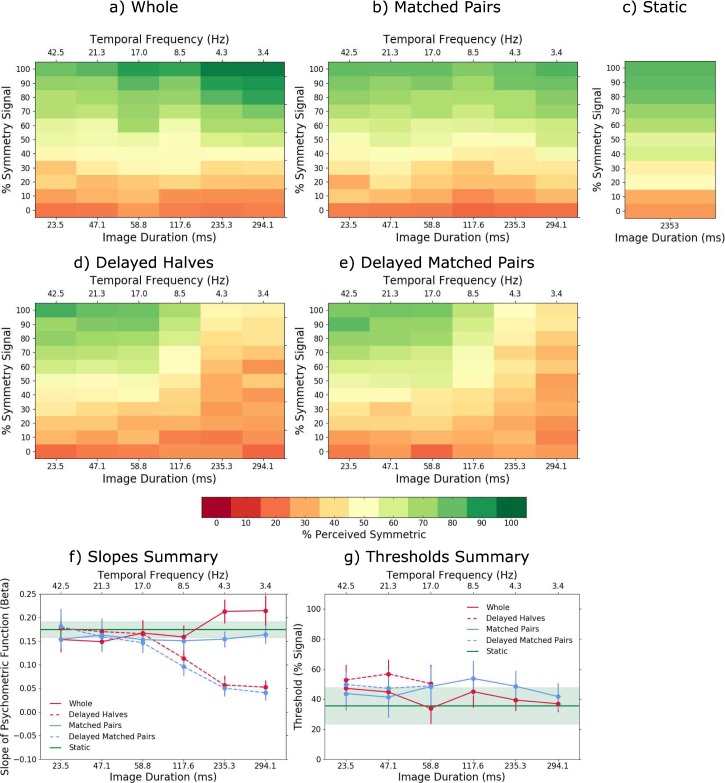
Sustained stimulus presentation experiment. The average across-observers percentage perceived symmetric as a function of the amount of stimulus symmetry (percentage symmetry signal) and image duration for (a) the whole patterns, (b) matched-pairs, (c) static, (d) delayed halves, and (e) delayed matched-pairs stimulus conditions. For clarity, we also show the temporal frequency (in Hz) corresponding to the two alternating images (see top horizontal axis). The color bar/key (below) shows the colors corresponding to each percentage perceived symmetric. The line graphs show averaged across-participants (f) slopes and (g) thresholds of the psychometric function for the whole patterns (red solid line), delayed halves (red dashed line), matched-pairs (blue solid line), delayed matched-pairs (blue dashed line) and static (green line). Errors bars and the green band for the static condition are ±1 SEM.

**Figure 3:**
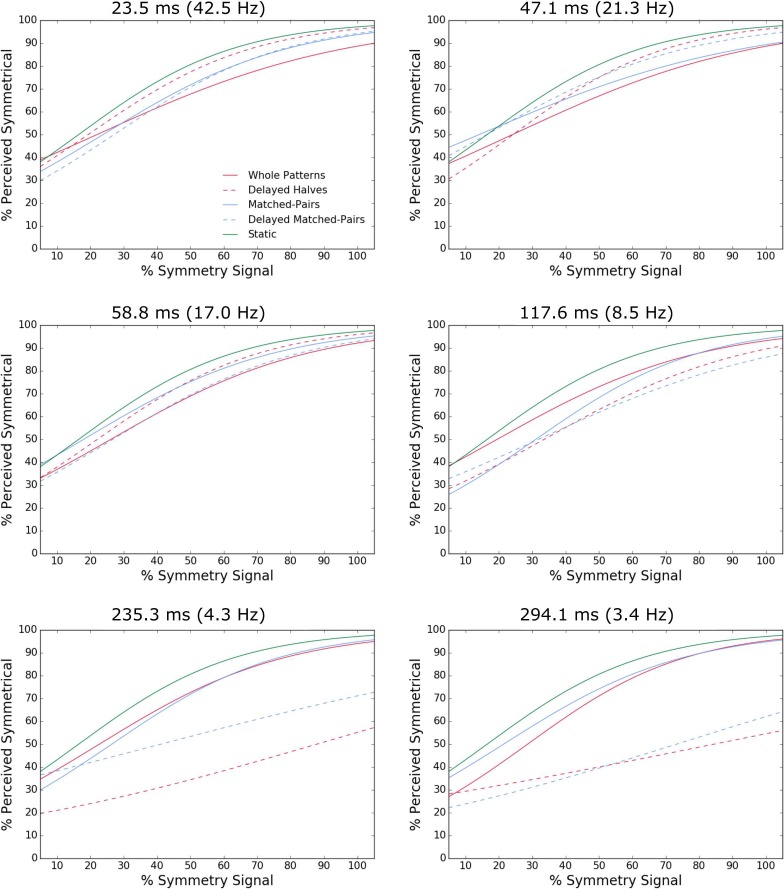
Example psychometric functions fitted to the percentage perceived symmetric data for one participant in the sustained stimulus presentation experiment. Logistic psychometric functions for the whole patterns (red solid line), delayed halves (red dashed line), matched-pairs (blue solid line), delayed matched-pairs (blue dashed line), and static (green line) conditions for each of the six image durations tested.

**Figure 4:**
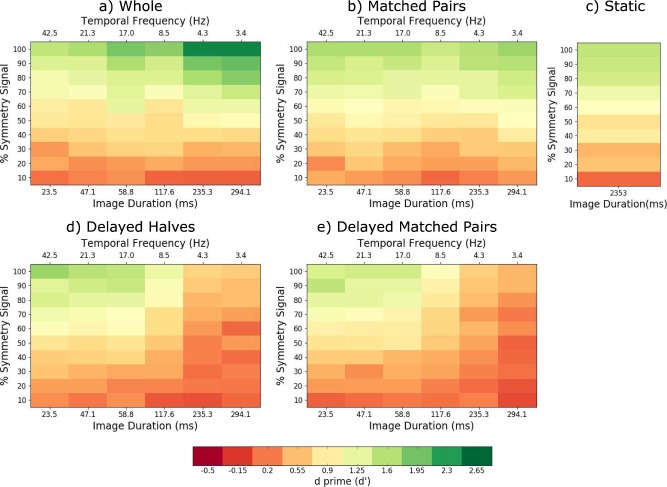
The d′ values for the sustained stimulus presentation experiment. The average across-observers d′ values for each amount of stimulus symmetry (percentage symmetry signal) and image duration/temporal frequency for (a) the whole patterns, (b) matched-pairs, (c) static, (d) delayed halves, and (e) delayed matched-pairs stimulus conditions. The color bar/key (below) shows the colors corresponding to each d′ value.

Figure 2 shows the average across-observers sensitivity (percentage perceived symmetric) in the symmetry perception task, as a function of the amount of symmetry in the stimulus (percentage symmetry signal) and image duration for the whole patterns (Figure 2a), matched-pairs (Figure 2b), static (Figure 2c), delayed halves (Figure 2d), and delayed matched-pairs (Figure 2e) conditions. For clarity, we also showed the temporal frequency (in Hz) corresponding to the two alternating images (see top horizontal axis). The green areas in Figure 2 indicate combinations of image duration (or temporal frequency) and percentage symmetry signal in the stimulus for which the observers perceived symmetry, and orange/red areas indicate that observers perceived no symmetry. The slope and threshold of the psychometric function corresponding to each temporal-arrangement condition are shown in Figure 2f and 2g, respectively. Example psychometric functions for each temporal arrangement condition and image duration (or temporal frequency) are shown in Figure 3 for one participant. The average across-participants *d′* values corresponding to the data in Figure 2 are shown in Figure 4 for each temporal arrangement condition.

The results in Figure 2 show that (a) for the whole-pattern condition, the percentage perceived symmetric increases with image duration when symmetry signal is larger than about 60% (compare lighter green areas for shorter image durations with darker green areas for longer image durations in Figure 2a). This is also reflected by the slopes *β* and thresholds of the psychometric functions fitted for each image duration (red lines in Figure 2f and g); (b) for the matched-pairs condition, the percentage perceived symmetric does not change with image duration (Figure 2b); thus, both the slopes (blue lines in Figure 2f) and the thresholds (blue line in Figure 2g) were comparable across image durations; (c) for both delayed halves and delayed matched-pairs conditions, the percentage perceived symmetric was similar and decreased gradually with image duration (Figure 2d and e), reaching the 75% level only for short (<60 ms) durations (dashed lines in Figure 2g). For durations longer than 60 ms, symmetry was hardly perceived, and hence thresholds were not possible to be estimated (see dashed lines in Figure 2g and also Figure 3). This is also seen in the slope of the psychometric function which decreases gradually with increasing image duration (dashed lines in Figure 2f); (d) with static stimuli, the percentage perceived symmetric was comparable to the whole and matched-pairs conditions and, with the delayed conditions but only for short (<60 ms) image durations. Thus, our results indicate that symmetry detection in dynamic stimuli is processed by high-pass temporal mechanisms, which are able to compute correlations across-the-symmetry-midline between symmetric pairs presented with temporal delays shorter than ∼60 ms (i.e., temporal frequencies higher than ∼17 Hz).

The *d*′ results shown in Figure 4 show a similar trend to the percentage perceived symmetric data shown Figure 2. As an indication, the average false alarm rates from which these *d*′ values were calculated were 0.23 for the whole pattern, 0.225 for the delayed halves, 0.212 for the matched-pairs, 0.253 for the delayed matched-pair, and 0.268 for the static pattern conditions. The range of *d*′ values obtained in this experiment is comparable to that found in previous studies that measured symmetry detection with static patterns (e.g., Barlow & Reeves, 1979, *d*′ = 0.8 − 1.2, approximately; Wenderoth, 1996b, *d*′ = 0.85 − 1.3, approximately).

A two-way repeated measures analysis of variance (ANOVA) with factors image duration (23.5, 47.1, 58.8, 117.7, 235.3, and 294.1 ms) and temporal arrangement (whole patterns, matched-pairs, delayed halves, and delayed matched-pairs) on the slope *β* data (Figure 2f) showed a significant main effect of image duration, *F*(5, 20) = 9.523, *p* < 0.0001, *η*^2^ = 0.0796, and temporal arrangement, *F*(3, 12) = 13.65, *p* = 0.0004, *η*^2^ = 0.1266, and, a significant interaction effect between image duration and temporal arrangement, *F*(15, 60) = 15.51, *p* < 0.0001, *η*^2^ = 0.2299. Bonferroni-corrected posthoc analysis showed that all pairwise comparisons between longer image durations (>60 ms) in the whole pattern and the delayed halves conditions were statistically significant (*p* < 0.05). Similarly, pairwise comparisons between longer image durations in simultaneous and delayed matched-pairs conditions were significant (*p* < 0.05). However, for the shorter image durations (<60 ms), none of the pairwise comparisons were significant (*p* > 0.05).

For the threshold data (Figure 2g), a two-way repeated measures ANOVA with factors image duration and simultaneous arrangement (whole patterns vs. matched-pairs) revealed no significant effect of image duration, *F*(5, 24) = 0.0928, *p* = 0.993, *η*^2^ = 0.0172, simultaneous arrangement, *F*(1, 24) = 3.908, *p* = 0.0597, *η*^2^ = 0.0105, or interaction effect, *F*(5, 24) = 1.367, *p* = 0.271, *η*^2^ = 0.0183. Similarly, the thresholds for the delayed-halves and delayed matched-pair conditions under short image durations (dashed lines in Figure 2g) were also not significant (*p* > 0.05).

### Transient stimulus presentation experiment

**Figure 5:**
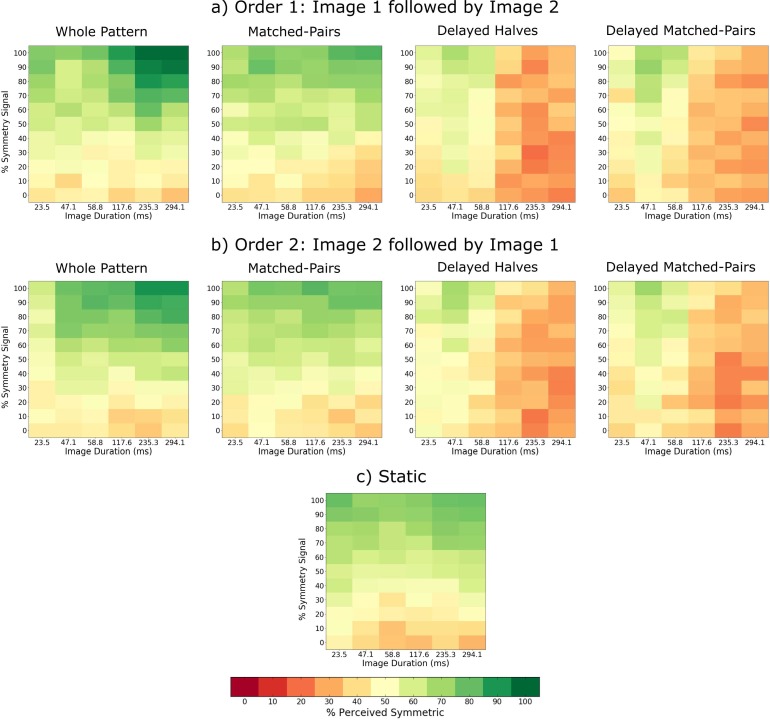
Transient stimulus presentation experiment. The average across-observers percentage perceived symmetric as a function of the amount of stimulus symmetry (percentage symmetry signal) and image duration for (a) Order 1: Image 1 followed by Image 2, (b) Order 2: Image 2 followed by Image 1, and (c) static conditions. The left-to-right panels indicate the results for the whole patterns, matched-pairs, delayed halves, and delayed matched-pairs conditions, respectively. The horizontal color bar at the bottom shows the colors corresponding to each percentage perceived symmetric.

**Figure 6:**
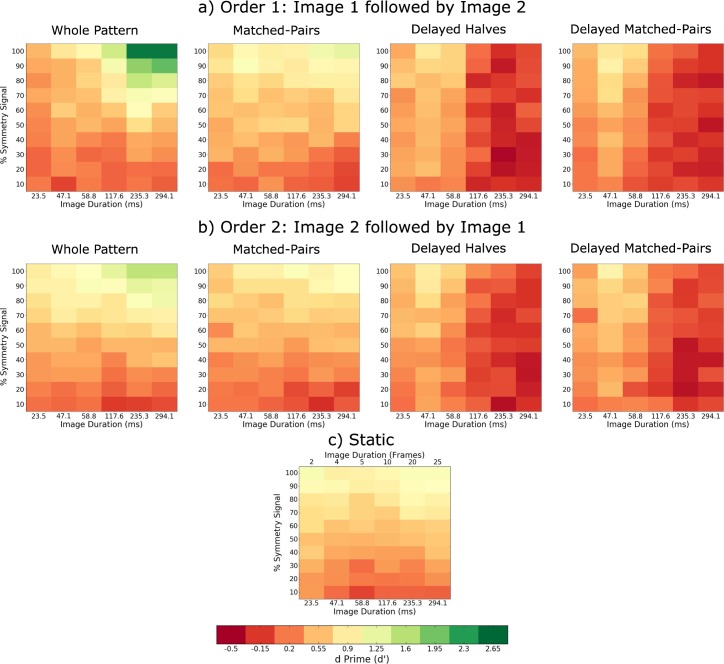
The d′ for transient stimulus presentation experiment. The average across-observers d′ values for each amount of stimulus symmetry (percentage symmetry signal) and image durations for (a) Order 1: Image 1 followed by Image 2, (b) Order 2: Image 2 followed by Image 1, and (c) static conditions. The left-to-right panels indicate the results for the whole patterns, matched-pairs, delayed halves, and delayed matched-pairs conditions, respectively. The horizontal color bar at the bottom shows the colors corresponding to each d′ value.

**Figure 7:**
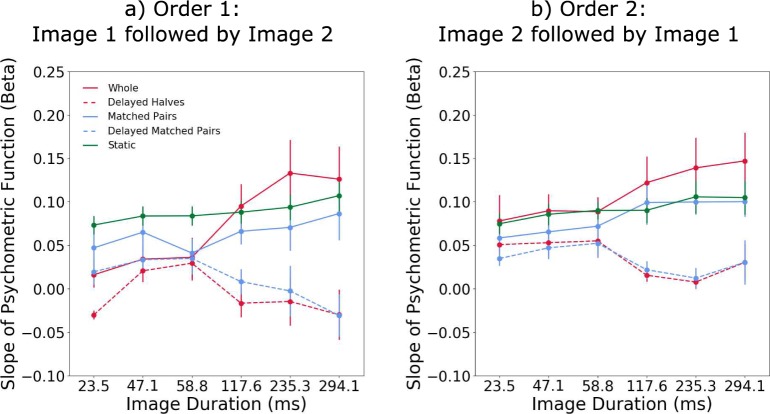
Slopes of the psychometric functions for the transient stimulus presentation experiment. The line graphs show the averaged across-participants slopes β as a function of image duration for (a) Order 1 and (b) Order 2 for the whole image (red solid line), delayed halves (red dashed line), matched pairs (blue solid line), delayed matched pairs (blue dashed line), and static (green line). Errors bars are ±1 SEM.

The percentage perceived symmetric results for transient stimulus presentation are shown in Figure 5 for order 1 (Figure 5a), order 2 (Figure 5b) and static (Figure 5c) conditions. As with the sustained conditions, we calculated *d*′ values for each observer and stimulus symmetry condition. The average across-observers *d*′ values are shown in Figure 6. The average across-observers false alarm rates for order 1 and order 2 were 0.416 and 0.4158 for whole patterns, 0.351 and 0.3475 for delayed halves, 0.3817 and 0.423 for matched-pairs, 0.3675 and 0.349 for delayed matched-pairs conditions 0.372 for the static condition. The slopes of the psychometric functions corresponding to the two temporal orders are shown in Figure 7. On average, these results follow a similar trend to those obtained with sustained stimulus presentation (Figure 2f) but the values for the slope *β* are lower by a factor of three, reflecting overall lower sensitivity to symmetry. For the whole pattern condition at longer image durations, sensitivity was slightly increased when the symmetrical image was presented before the noise image (i.e., order 1 or backward masking) than vice-versa (order 2 or forward masking) condition—compare the first panel in Figure 5a with Figure 5b.

The data (slope *β*) for each presentation order condition were separately submitted to a two-way repeated measures ANOVA, with factors image duration (23.5, 47.1, 58.8, 117.7, 235.3, and 294.1 ms) and temporal arrangement (whole pattern, matched-pairs, delayed halves, delayed matched-pairs, and static). The analysis revealed a significant main effect of image duration for order 2, *F*(5, 15) = 4.776, *p* = 0.0082, *η*^2^ = 0.0454, but not order 1, *F*(5, 15) = 2.074, *p* = 0.1258, *η*^2^ = 0.0156, conditions. The main effect of temporal arrangement was found to be statistically significant for both order 1, *F*(4, 12) = 15.75, *p* = 0.0001, *η*^2^ = 0.3217, and order 2, *F*(4, 12) = 15.45, *p* = 0.0001, *η*^2^ = 0.3065, conditions. The interaction effect between image duration and temporal arrangement was also significant for both order 1, *F*(20, 60) = 3.558, *p* < 0.0001, *η*^2^ = 0.09523, and order 2, *F*(20, 60) = 6.887, *p* < 0.0001, *η*^2^ = 0.2126, conditions. Bonferroni-corrected posthoc analysis showed a comparable pattern of statistically significant pairwise comparisons to the sustained condition with the following exceptions: In order 1, the whole pattern condition with short (23.5 ms) image duration was significantly different from image durations longer than 117.7 ms, and the delayed halves condition with short (23.5 ms) image duration was significantly different from the synchronous conditions with longer image durations (>60 ms). In order 2, the matched-pairs condition was also significantly different to the whole pattern condition with longer image durations (>60 ms) and to the longest static conditions (235.3 and 294.1 ms).

### Comparison between transient and sustained presentations

In order to determine whether sensitivity differed between the transient and sustained conditions, we used a two-way repeated-measures ANOVA on the slope of the linear regression line that relates the β parameter of the psychometric function to image duration for each stimulus presentation type (sustained vs. transient order 1 vs. transient order 2) and temporal arrangement conditions (whole patterns, delayed halves, matched-pairs, and delayed matched-pairs). The analysis revealed a significant main effects of presentation type, *F*(3, 12) = 30.73, *p* < 0.0001, *η*^2^ = 68.38, and temporal arrangement, *F*(2, 12) = 10.72, *p* = 0.0021, *η*^2^ = 15.9. This significant difference between sustained and transient stimulus presentation appears to be driven by the delayed conditions which have shallower linear regression slopes in the transient than in the sustained conditions. There was no significant interaction between presentation type and temporal arrangement.

## Discussion

We have examined symmetry perception in response to sustained and transient presentations of dynamic patterns using different temporal arrangements of symmetrical and random elements. Our results show that (a) with on-going, sustained presentations of symmetrical and noise patterns (i.e., whole pattern condition), sensitivity increased significantly for image durations longer than about 120 ms; (b) for the delayed conditions, when the symmetrical pairs or halves were presented in different temporal intervals, sensitivity decreased gradually with image durations longer than ∼60 ms, suggesting that symmetry detection mechanisms can tolerate time delays between positional symmetric-elements of up to 60 ms; (c) for the sustained presentation of symmetric patterns containing 50% symmetric pairs, sensitivity was invariant with image duration when the two alternating images contained equal amounts of symmetry; (d) for the transient presentation, sensitivity gradually improved as a function of image duration when the two images contained equal amounts of symmetry or when they were the static, time-averaged patterns; (e) on average, sensitivity was higher when the symmetric image preceded (i.e., backward masking) rather than followed (i.e., forward masking) the noise pattern. Altogether, the results for the whole and delayed conditions indicate that spatial-correlation across the symmetry axis can be integrated over time within ∼120 ms time window and consequently symmetry mechanisms can tolerate delays of up to 60 ms.

Sensitivity to symmetry in the whole pattern condition increased significantly for image durations longer than 120 ms (see green areas in Figure 4a and 6a), suggesting that symmetry detection mechanisms integrate symmetric and noise patterns within a time window of ∼120 ms. For sustained stimulus presentation, we found comparable sensitivity to symmetry between simultaneous and delayed image conditions up to about 60 ms image duration (see green areas in Figure 2d and also thresholds in Figure 2g), suggesting that symmetry detection mechanisms can compute spatial correlations between temporally delayed matched dot-pairs and/or between symmetric halves of up to 60 ms. This tolerance for temporal delays of up to 60 ms is a consequence of a temporal integration process occurring within 120 ms. The present results show that the computation of spatial correlations across-the-symmetry-midline over time is limited to about 17 Hz alternation frequency, suggesting that symmetry detection in dynamic stimuli is processed by a relatively high-pass temporal mechanism.

In our experiments, we found increased sensitivity with sustained compared to transient stimulus presentations suggesting that symmetry mechanisms integrate simultaneously presented matched-pairs over time. Overall, the slopes *β* of the psychometric function for the transient conditions were about three times lower than for the sustained presentation conditions (compare Figure 2f and Figure 7). Additionally, the slopes of the linear regression lines are shallower for sustained presentation compared to transient presentation, suggesting that sensitivity decreases more rapidly with image duration when presentation is transient. This lower sensitivity with transient stimuli comes at odds with previous literature showing that symmetry can be reliably perceived in very briefly presented stimuli of under 50 ms (Julesz, 1971; Tyler et al., 1995). However, a number of studies have shown that even with fully symmetric patterns (100% symmetry signal), performance never reached 100% correct detection but remains limited to ∼80% correct detection (Tyler et al., 1995; Wenderoth, 1996a). For the whole patterns in the transient presentation condition, sensitivity to symmetry was affected by the presentation order of the symmetric and noise patterns, with significantly lower sensitivity for shorter image durations (less than 60 ms) when the symmetrical pattern was presented before the noise pattern (i.e., backward masking). This is similar to findings from depth perception studies where with transient stimulus presentations the perception of stereoscopic depth was affected by the presentation order of correlated and uncorrelated random dot images (Gheorghiu & Erkelens, 2004).

Our results showing that symmetry can be perceived in delayed halves and delayed matched-pairs conditions for very short image durations (<60 ms), despite there being no symmetrical matched-pairs in either time interval, suggest that symmetry detection mechanisms can compute spatial correlation across the symmetry axis between matched pairs presented with short delays and integrate these weak symmetry signals over a time period of ∼120 ms. The tolerance to delays between presentations of matched-pairs up to ∼60 ms found in the current study is in keeping with previous findings by Niimi, Watanabe, & Yokosawa (2005) who reported that symmetry can be detected in briefly-presented split symmetric-halves presented with a stimulus onset asynchrony (SOA) of 87 ms. These authors explained their results in terms of visual persistence (i.e., a briefly presented stimulus outlasts its physical presentation on the screen) or visual memory (Di Lollo, 1980; Niimi et al., 2005) given that the images were briefly flashed for 13 ms. However, by using a variable stimulus onset asynchrony (SOA), the strength of briefly presented symmetry signals may decay over time at different rates depending on image duration, and one cannot define temporal frequency for unequal combinations of image durations and SOAs.

Altogether our sustained and transient presentation results suggest that symmetry mechanisms can integrate weak symmetry signals over a time period of 120 ms. The lower sensitivity with transient compared to sustained stimulus presentations could be explained by the presence of a weaker, transient symmetry signal within the temporal integration period than when the symmetry signals are presented in an ongoing manner. This explanation is in keeping with previous findings from Cohen and Zaidi (2013) showing that the temporal thresholds for detecting the orientation of the axis of symmetry in natural textures varied broadly, suggesting a wide range of stimulus salience as quantified by the inverse of the temporal threshold (i.e., 1/threshold).

Recently, Cohen and Zaidi (2013) have proposed a model for estimating symmetry-energy in natural textures by connecting pairs of symmetric spatial filters simulating the RFs of neurons. If the two orientations were related by mirror-symmetry, then an AND junction was activated. If the outputs of the two filters were about equal, then they were summed into a symmetry-energy index which accurately identified the spatial position of the axis of symmetry for most stimuli but correlated poorly with the stimulus salience (i.e., 1/temporal-threshold). Thus, it remains unclear what consequences time (i.e., duration and synchronization of symmetric pairs) has on this model as well as on other models of symmetry detection based on spatial oriented filters (Dakin & Watt, 1994; Rainville & Kingdom, 2002). However, our findings suggest that the current models of symmetry detection (e.g., the AND-gating model of Cohen & Zaidi, 2013) must include computations of spatial correlations between the outputs of spatiotemporal oriented filters that integrate symmetry information within ∼120 ms. If the outputs of the two filters are delayed longer than 60 ms, then the AND-gate will not be activated and symmetry will not be perceived.

Due to the long overall stimulus duration in the sustained presentation experiment (2.35 s), one might think that eye movements could contribute to symmetry detection (Meso, Montagnini, Bell, & Masson, 2016). Meso et al. (2016) reported that eye movements made by observers viewing static symmetric stimuli generated more saccades parallel to the axis of symmetry than along other orientations, and this observed parallel orientation-selectivity emerged within 500 ms of stimulus onset. Although our sustained stimulus presentation was 2.35 s, it is unlikely that eye movements contributed to our results as each image was only presented briefly, for between 23.5 and 294.1 ms. These image durations are shorter than the time needed to plan eye movements (<180–200 ms; Collewijn, Erkelens, & Steinman, 1997) and/or scan the images (Meso et al., 2016).

### Relationship with electrophysiological and neurophysiological studies

A number of studies examined the time course of neuronal responses to symmetry perception by measuring event related potentials (ERP) in response to symmetric and quasirandom patterns (Bertamini & Makin, 2014; Wright, Mitchell, Dering, & Gheorghiu, 2018). These studies found that the amplitude in posterior electrodes is comparable for symmetric and quasirandom patterns up to 200 ms after stimulus onset. After that time (i.e., 200–600 ms) the amplitude becomes lower for symmetric than quasirandom patterns, resulting in a difference-wave termed the Sustained Posterior Negativity (SPN; Bertamini & Makin, 2014; Norcia, Candy, Pettet, Vildavski, & Tyler, 2002). These studies suggest that symmetry is extracted relatively late, after nonsymmetric specific form processing (Norcia et al., 2002). The current work does not address the time course of neuronal/electrophysiological responses to symmetric stimuli but rather examined the temporal properties of symmetry perception by considering how temporal synchrony/asynchrony between matched pairs and image duration affect the integration of perceptual grouping of symmetrical elements across the vertical axis over time. This differs from ERP findings, as the SPN is not necessarily related to symmetry per se, but rather structure or regularity in a stimulus (Bertamini & Makin, 2014) and, therefore, may not reflect the temporal accumulation or integration process required to perceive symmetry.

Neuro-imaging studies have shown that symmetry generates a distinctive pattern of brain activity over a wide network of extrastriate areas (Sasaki et al., 2005; Tyler et al., 2005). To our knowledge, there are no neurophysiological studies of symmetry perception in neurons sensitive to symmetry. Although brain imaging studies found that there is no differential activation in areas V1 and V2 for symmetrical versus asymmetrical stimuli (Cattaneo, Mattavelli, Papagno, Herbert, & Silvanto, 2011; Chen, Kao, & Tyler, 2007; Sasaki et al., 2005), there is some neurophysiological evidence that V1 neurons exhibit enhanced responses at the medial (symmetry) axis of simple geometric figures defined by texture, about 80 ms after stimulus onset (Lee, Mumford, Romero, & Lamme, 1998). However, it is unclear what the consequences of temporal delays are for neurons exhibiting sensitivity to the medial axis of symmetry. It is known that symmetry is poor in the periphery (Gurnsey et al., 1998) and perception is focused around the axis of symmetry with the exact size of the *spatial* integration window determined by the size of pattern elements (Rainville & Kingdom, 2002). However, direct neurophysiological research is needed to understand the dynamics of symmetry mechanisms at neuronal level.

To conclude, we showed that observers' sensitivity to symmetry was higher for sustained compared to transient presentations and when symmetrical pairs were presented simultaneously rather than with temporal delay. Overall, we found (a) comparable sensitivities between simultaneous and delayed conditions up to about 60 ms per image, suggesting that symmetry signals are integrated over a time period of ∼120 ms and (b) a gradual decrease in sensitivity in the delayed conditions for longer (>60 ms) image durations. We conclude that spatial correlation between matched-pairs (and/or stimulus halves) across the symmetry axis can be integrated over time, and symmetry detection mechanisms can tolerate temporal delays between symmetrical pairs of up to approximately 60 ms.

## Supplementary Material

Supplement 1

Supplement 2

Supplement 3

Supplement 4

## Supplementary material

*Supplementary Movie S1*. Whole patterns condition, in which a symmetrical pattern is alternated with a noise pattern.

*Supplementary Movie S2*. Delayed halves condition, in which left and right halves of the symmetrical and noise patterns were presented with temporal delay, that is, half of the symmetrical pattern is presented in each interval.

*Supplementary Movie S3*. Matched-pairs condition, consists of two alternating symmetrical patterns each containing equal amounts of symmetrical matched-pairs. Each individual image (or interval) contained half of the total number of symmetrical dots.

*Supplementary Movie S4*. Delayed matched-pairs condition is the same as the matched-pairs condition but with the matched-pairs presented in different intervals, that is, with temporal delay.
