# Supplementary figures and images for: Temporal dynamics of mirror-symmetry perception
Source: J Vis. 2018 May 15;18(5):10. doi: 10.1167/18.5.10 (PMC5954970; doi:10.1167/18.5.10)

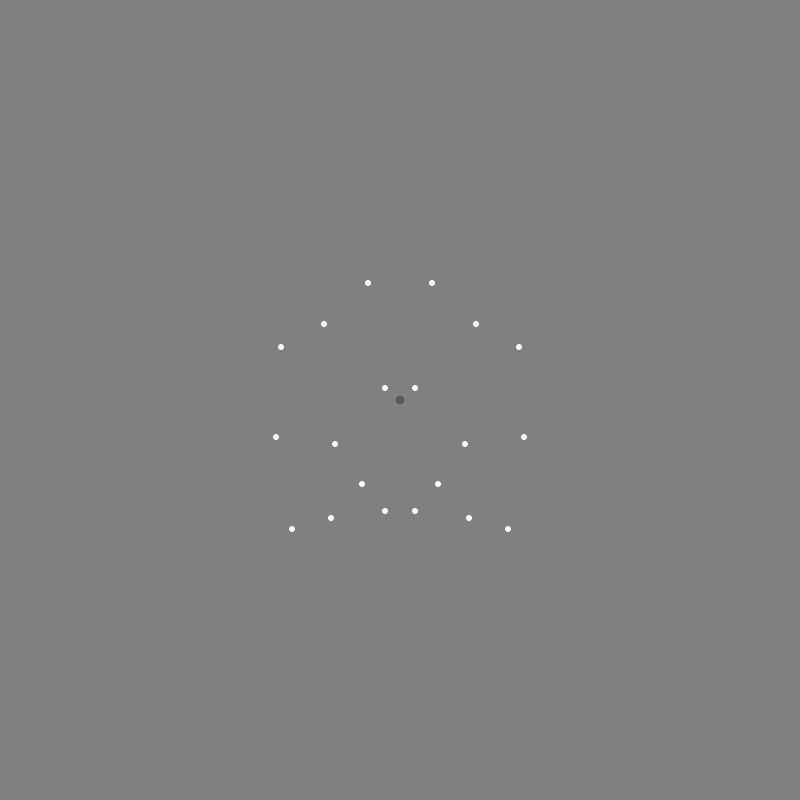

Supplement: Supplement 1 [file jovi-18-05-03_s01.gif]

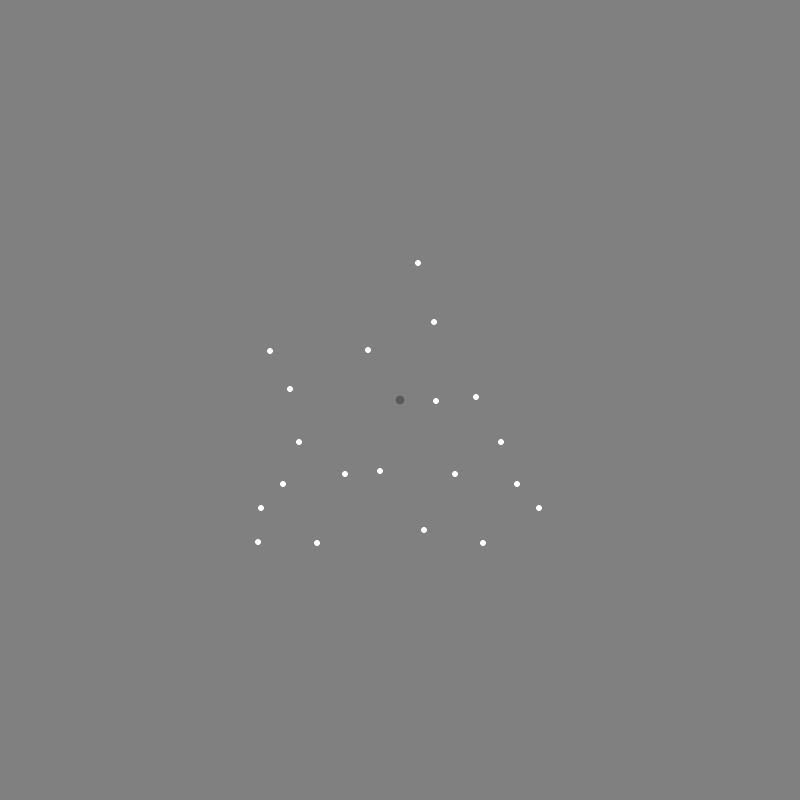

Supplement: Supplement 2 [file jovi-18-05-03_s02.gif]

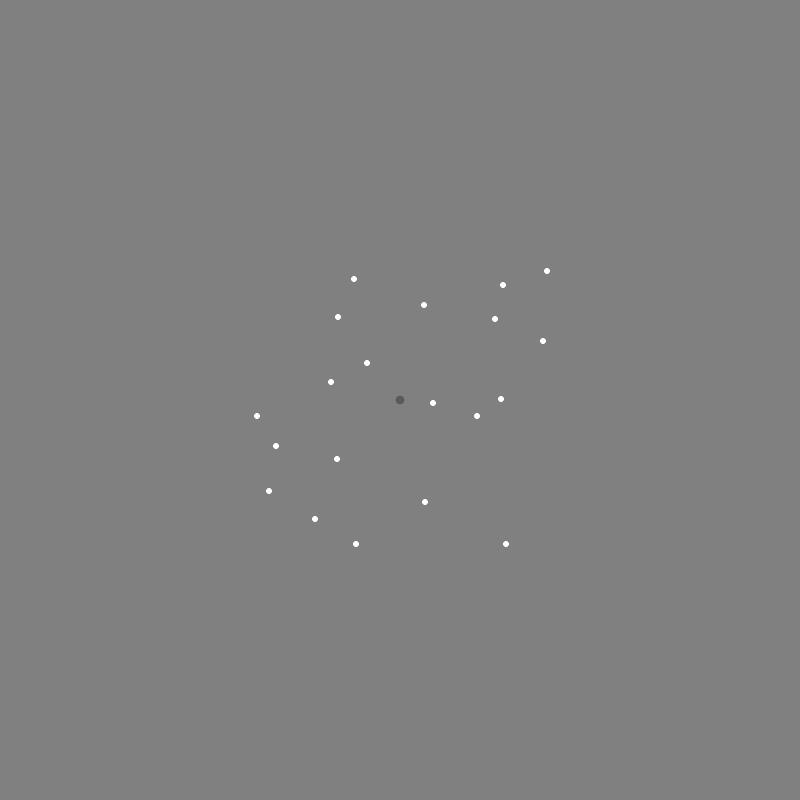

Supplement: Supplement 3 [file jovi-18-05-03_s03.gif]

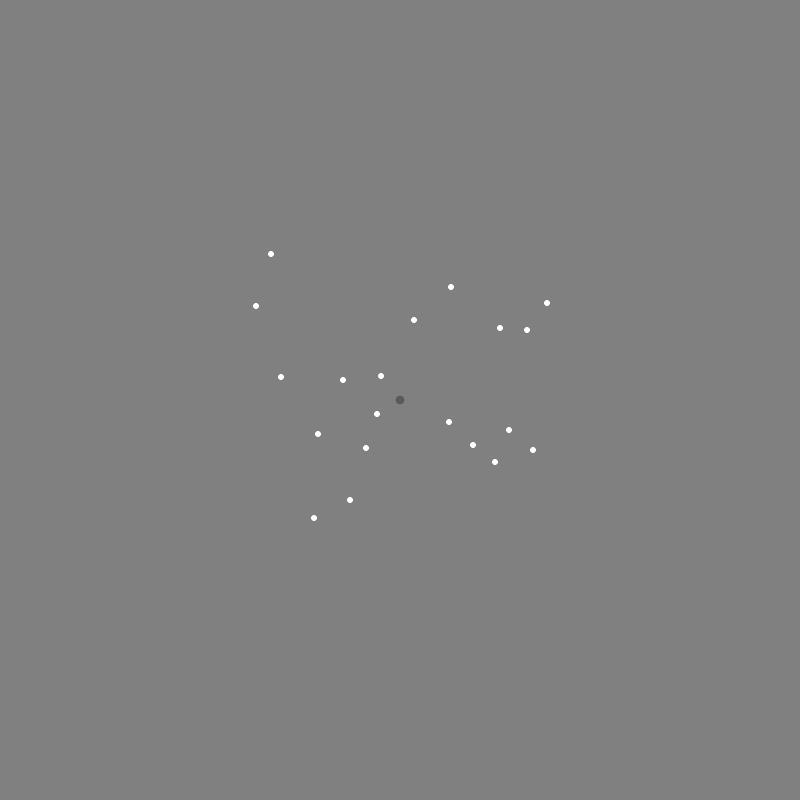

Supplement: Supplement 4 [file jovi-18-05-03_s04.gif]
